# Thermal and Mechanical Properties of Bamboo Fiber Reinforced Epoxy Composites

**DOI:** 10.3390/polym10060608

**Published:** 2018-06-03

**Authors:** Kai Zhang, Fangxin Wang, Wenyan Liang, Zhenqing Wang, Zhiwei Duan, Bin Yang

**Affiliations:** 1 College of Aerospace and Civil Engineering, Harbin Engineering University, Harbin 150001, China; zhangkai24@hrbeu.edu.cn (K.Z.); Wangfangxin@hrbeu.edu.cn (F.W.); wangzhenqing@hrbeu.edu.cn (Z.W.); duanzhiwei@hrbeu.edu.cn (Z.D.); 2 School of Mechanical and Power Engineering, East China University of Science and Technology, Shanghai 200237, China; yangbin@ecust.edu.cn

**Keywords:** fiber-matrix composites (FMCs), alkali treated bamboo fiber, mechanical properties, thermal properties

## Abstract

Bamboo fibers demonstrate enormous potential as the reinforcement phase in composite materials. In this study, in order to find suitable NaOH concentration for bamboo fiber treatment, bamboo fibers were treated with 2 wt.%, 6 wt.% and 10 wt.% NaOH solutions for 12 h, respectively. We determined that 6 wt.% NaOH treated bamboo fibers were optimal for the fabrication of bamboo fiber composites by single fiber tensile test, single fiber pull-out test, Fourier transform infrared spectroscopy (FTIR), and scanning electron microscopy (SEM). The short length bamboo fibers treated with 6 wt.% NaOH solutions were well dispersed in the epoxy matrix by a new preparation method. The effect of fiber content and fiber length on the mechanical behavior of bamboo fiber reinforced epoxy composites was investigated. The results confirmed that fracture toughness and flexural modulus of the composites monotonically increased with fiber length and content. However, for all samples, composites showed negligible difference on the flexural strength. The fracture surfaces of the composites were observed by SEM, revealing that fiber breakage, matrix cracking, debonding, and fiber pull out were major failure types. In addition, thermogravimetric analysis (TGA) was carried out to investigate the thermal behavior of both bamboo fibers and composites.

## 1. Introduction

From the 21st century, due to the serious issues of environmental pollution and energy shortages, biomass composites, including natural-fiber-reinforced composites, have received great attention from scientists and researchers [1]. In the reinforcing phase, plant fibers such as sisal, kenaf, banana, pennisetum purpureum, and bamboo were successfully applied in both thermoplastic and thermosets matrices [2,3,4,5,6]. Natural fibers have many appealing characteristics as low cost, low density, low energy requirements, abundant availability, renewability, no skin irritation, higher strength to weight ratio, high aspect ratio (L/D), and high strength and elasticity modulus, thus showing great potential as replacements of glass, carbon, or other synthetic fibers [7,8]. According to Ratna Prasad and Mohana Rao [9], bamboo fibers possessed relatively higher ultimate tensile strength than most of plant fibers, such as jowar and sisal. As the kingdom of bamboo, China was the most famous country in the world, with more than 500 species of bamboo and an area of bamboo forest of around 67,000 hectares, accounting for 1/4 of the world’s bamboo forests [10]. Nevertheless, the utilization rate of natural bamboo resources was still very low, only about 40% [11].

Meanwhile, due to the hydrophilic nature of plant fibers and hydrophobic nature of resins, compatibility problems limited the further application of natural fiber composites [12]. Natural fiber composites without any pre-treatment have poor interfacial adhesion between natural fibers and matrix resins; this often results in the creation of porous materials, environmental degradation, and the deterioration of mechanical properties. Hence, substantial work has been reported to improve the interfacial adhesion by physical, chemical, or other modification methods. Common surface modification methods included alkali treatment, acetylation, cyanothylation, and coupling agent treatment, among which alkali treatment was determined to be one of the simplest and most effective methods [13,14,15,16,17].

Epoxy resin (EP), as one of the most commonly-used thermosets, has been widely applied in various engineering fields due to its excellent mechanical properties, chemical resistance, and electrical insulation [18]. However, EP also has many shortcomings, such as poor fracture performance and too much sensitivity to cracks [19]. Hence, a large amount of research has been done to tailor the mechanical properties of EP by adding inorganic-particulate fillers or synthetic fibers [20,21,22,23]. Meanwhile, several researchers tried to add natural fibers to EP for a more cost-effective and environment-friendly composite. Lu et al. [16] cut bamboo fibers into bamboo cellulose powder using a high speed universal grinder, and fabricated cellulose/epoxy composites by casting method. Lu et al. [16] confirmed that NaOH treated and KH560 treated cellulose fiber reinforced epoxy composites apparently exhibited better mechanical properties than unmodified ones. In another research, Khan et al. [24] prepared bamboo-fiber-reinforced epoxy composites by the traditional hand lay-up method, and found that composites having 25 mm fiber length had greater fracture toughness values (K_IC_) than composites with smaller fiber lengths. Similarly, Rosa et al. [25] investigated tensile and flexural behavior of short and quasi-unidirectional New Zealand flax-fiber-reinforced epoxy composites, and determined that only quasi-unidirectional fiber reinforced composites showed better mechanical properties than neat epoxy. In spite of this, till now, the effect of fiber content and length on flexural and fracture properties of bamboo fiber reinforced epoxy composites has not been thoroughly studied.

In this study, 6 wt.% NaOH treated bamboo fibers were proven to be optimum for the fabrication of bamboo fiber composites by single fiber tensile test, single fiber pull-out test, Fourier transform infrared spectroscopy (FTIR), and scanning electron microscopy (SEM). A comprehensive study has been done on the effect of fiber content and length on flexural and fracture behavior of bamboo fiber reinforced epoxy composites. In addition, the thermal behavior of bamboo fibers and their composites was also investigated.

## 2. Experimental Section

### 2.1. Materials

The bamboo-fiber-reinforced epoxy composites consisted of 6 wt.% NaOH-treated natural bamboo fibers and epoxy resin. Bamboo fibers were kindly donated by a local textile company located at Ningbo city of Zhejiang province in East China. Diglycidyl ether of bisphenol-A (DGEBA, YD-128, Kukdo Chemical (Kunshan) Co., Ltd., Kunshan, China) and a kind of modified amine hardener (5010B, Tianjin Fan-Xing Electronic Materials Co., Ltd., Tianjin, China) were used as the epoxy compound and curing agent, respectively. This modified amine hardener was selected due to its low viscosity and slow curing rate.

### 2.2. Single Fiber Tensile Test

Firstly, bamboo fibers were manually sized into lengths of 5 mm, 10 mm and 15 mm, respectively. Secondly, fibers were soaked in 2 wt.%, 6 wt.% and 10 wt.% NaOH solutions at room temperature for 12 h. The mass ratio of NaOH solution and bamboo fibers was 20:1. After alkali treatment, fibers were washed thoroughly with clean water to neutralize pH, and dried in air for 24 h. Finally, fibers were placed in an oven for another 12 h at 60 °C, and then carefully stored in a sealed plastic bag.

**Figure 1:**
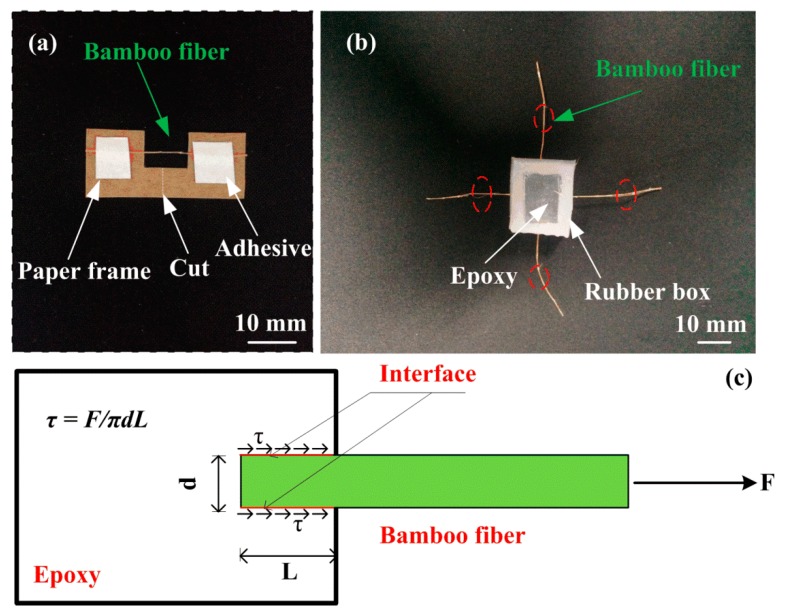
Schematic diagram illustrating: (**a**) Specimen for single fiber tensile test, (**b**,**c**) Schematic representation of the fiber pull-out specimen and test.

**Figure 2:**
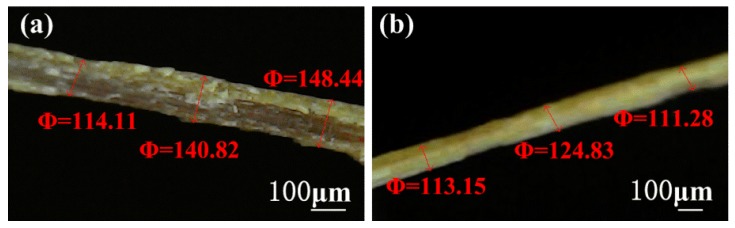
Measurement of diameters of untreated bamboo fiber (**a**) and 6 wt.% NaOH treated bamboo fiber (**b**) by optical microscope.

A single fiber tensile test was conducted on a Z-Wick Testing Machine, following the procedure described in [26], as shown in Figure 1a. The diameters of bamboo fibers were measured with an optical microscope (see Figure 2).

### 2.3. Single Fiber Pull-Out Test

As shown in Figure 1b,c, in order to determine the interfacial shear strength (IFSS) between a single bamboo fiber and epoxy, a single fiber pull-out test was carried out on a Z-Wick Testing Machine at a cross head speed of 1 mm/min. The detailed preparation of samples for single fiber pull-out test was discussed in Ref. [17]. The IFSS value is calculated as follows [27]:

(1) $$\tau =\frac{\mathrm{Failure}\;\mathrm{load}}{\mathrm{Interfacial}\;\mathrm{area}}=\frac{F_{\max}}{\pi \mathrm{dL}}$$

where F_max_ is the maximum force, d is the diameter of the bamboo fiber, and L is the embedded fiber length in the epoxy matrix.

### 2.4. Mechanical Properties

#### 2.4.1. Fabrication of Bamboo Fiber Reinforced Epoxy Composites

Bamboo fibers treated by 6 wt.% NaOH solutions were used for the preparation of bamboo fiber composites. Considering the difficulty of adding a high content of short bamboo fibers into the epoxy matrix, a new preparation process was developed. First, epoxy resin was heated in oven at 80 °C for approximately 15min to reduce the viscosity and remove the entrapped air. Meanwhile, bamboo fibers were placed in the oven to ensure complete drying. Second, compared with nano-micron-particles or powder fillers, short bamboo fibers are porous and have higher specific surface area. Hence, a lot of air was brought into the epoxy resin when the fibers were added, which could lead to the mechanical degradation of the resulting composites. Therefore, the amount of epoxy resin was increased by about 30 wt.% for better infiltration of fibers and the removal of bubbles. Third, the mixture was put into a vacuum drying oven at 80 °C for approximately 2 h. After sufficient infiltration of fibers and removal of bubbles, the low viscosity curing agent was added and carefully stirred, using glass rod, for 5 min. The mixture was then poured into a self-made groove mold. Finally, trapped air and redundant epoxy was gently squeezed out of the mixture using a flat-ended metal probe. The mass ratio of epoxy and hardener was 3:1. For comparison, neat epoxy was also prepared. All the samples were cured at room temperature for 24 h. Additionally, the weight fraction of the composites was calculated after complete curing. Prior to mechanical testing, all the samples were placed in room conditions for at least 7 days. The prepared bamboo-fiber-reinforced-epoxy composites are referred to as BF/EP_X_Y, where X denotes the bamboo fibers weight fraction and Y denotes fiber length.

#### 2.4.2. Flexural Testing

A universal Instron testing machine was used to conduct the flexural test at a crosshead speed of 0.1 mm/min. Composite specimens of dimension, 80 mm  ×  10 mm  ×  5 mm, were symmetrically loaded in a three-point-bend fixture along the thickness direction. Flexural modulus ($E_{f}$) and flexural stress ($\sigma_{f}$) were calculated by the following equations [28]:

(2) $$E_{f}=\frac{{\mathrm{mS}}^{3}}{{4\mathrm{WB}}^{3}}$$

where S, W, B, and m represent the sample span, width, thickness and the crosshead displacement, respectively.

(3) $$\sigma_{f}=\frac{3\mathrm{PS}}{{2\mathrm{WB}}^{2}}\left[1+6{\left(\frac{\delta }{S}\right)}^{2}-\left(\frac{B}{S}\right)\left(\frac{\delta }{S}\right)\right]$$

The bracketed term in Equation (3) was used due to the large S/B ratio of the specimens [28]. The load and deflection at the midspan of the beam are represented by P and δ, respectively.

#### 2.4.3. Quasi-Static Fracture Toughness Test

**Figure 3:**
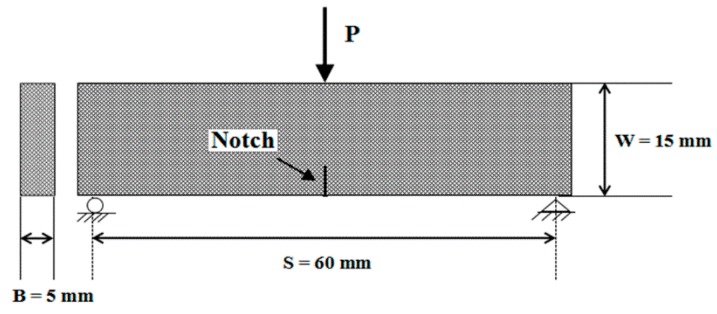
Single edge notched beam (SENB) specimen configuration for fracture experiments.

In this study, Linear Elastic Fracture Mechanics (LEFM) theory was used because of the overall brittle nature of the resulting composites [29]. As shown in Figure 3, single edge notched beam (SENB) specimens were used to conduct a quasi-static fracture toughness test, according to ASTM Standard D5045-99 [30]. An edge notch of 2 mm length was introduced at the middle span of each specimen of dimension of, 70 mm  ×  15 mm  ×  5 mm, using a diamond impregnated circular saw. Then, a natural crack was carefully initiated by a sharp razor blade. The ratio of the crack length to the specimen width (a/W) was between 0.45 and 0.55. According to Ref. [30], the SIF (K_I_) was calculated by the following equation:

(4) $$K_{I}=\frac{\mathrm{PS}}{{\mathrm{BW}}^{3/2}}\;f\left(\xi \right)$$

In the above equation, P and f(ξ) represent concentrated force applied in the middle of the sample and geometric factor. f(ξ) is given by:

(5) $$f\left(\xi \right)=\frac{3\sqrt{\xi }\left\{1.99-\xi \left(1-\xi \right)\left(2.15-3.93\xi +2{.7\xi }^{2}\right)\right\}}{2\left(1+2\xi \right){\left(1-\xi \right)}^{3/2}},\left(\xi =a/W\right)$$

P is replaced by the load corresponding to the fracture initiation for calculating mode-I fracture toughness K_IC_.

### 2.5. TGA/DTG

The thermal stability of bamboo fibers and bamboo-fiber-reinforced-epoxy composites was evaluated by thermogravimetry (TGA) and differential thermogravimetry (DTG) techniques (TGA Q50 V20.13 Build 39). The fiber samples weighed about 2 mg, while the composites samples weighed 10–15 mg. All samples were placed in a platinum pan and heated from 40 °C to 800 °C, at a heating rate of 20 °C/min, under a nitrogen atmosphere at a flow rate of 40 mL/min.

### 2.6. FTIR

In order to analyze the change of chemical structures of bamboo fibers after alkali treatment, Fourier transform infrared spectra was conducted by dispersing powdered bamboo fiber samples in KBr pellets (mass ratio; bamboo fiber:KBr = 1:100), using a Bio-Rad FTS 3000 MX spectrometer (Varian, Inc., Palo Alto, CA, USA). All spectra were recorded in the wave number range of 400–4000 cm^−1^ with resolution of 4 cm^−1^.

### 2.7. SEM

The surface morphologies of untreated and alkali-treated bamboo fibers, as well as the fracture-surface morphologies of bamboo fiber reinforced epoxy composites, were studied by scanning electron microscope (SEM, Zeiss MA15, Hitzacker, Germany). Before SEM characterization, each sample was uniformly coated with platinum and the SEM test was performed in the high vacuum mode at accelerating voltages of 10 kV.

## 3. Results & Discussion

### 3.1. SEM Images of Alkali-Treated Bamboo Fibers

**Figure 4:**
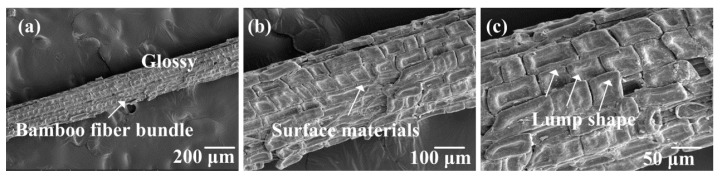
SEM images of the surface of the untreated bamboo fiber bundles.

**Figure 5:**
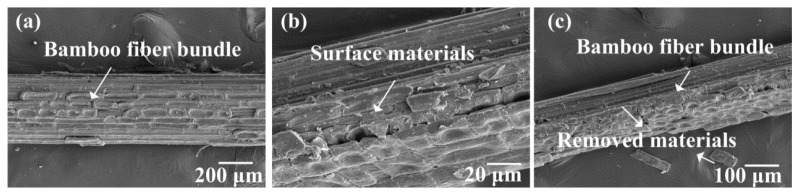
SEM images of the surface of the 2 wt.% NaOH-treated bamboo fiber bundles.

**Figure 6:**
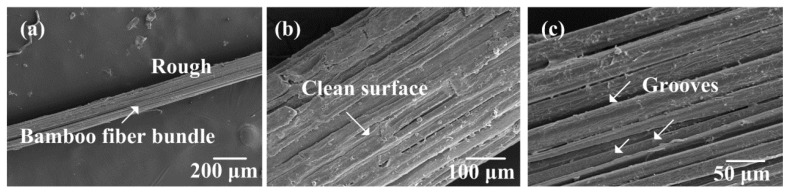
SEM images of the surface of the 6 wt.% NaOH-treated bamboo fiber bundles.

**Figure 7:**
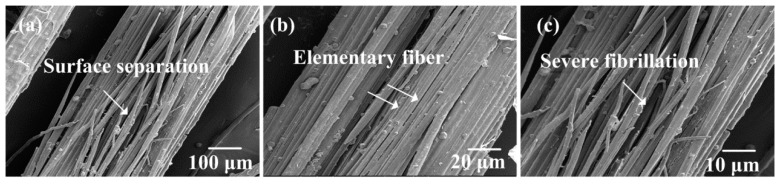
SEM images of the surface of the 10 wt.% NaOH-treated bamboo fiber bundles.

Figure 4, Figure 5, Figure 6 and Figure 7 show the surface morphologies of untreated and 2–10 wt.% NaOH treated bamboo fibers, respectively. It is evident that bamboo fibers treated with different concentrations of NaOH solutions display completely different surface morphologies.

As shown in Figure 4, the surface of untreated bamboo fibers were relatively smooth and covered with many lump materials. These surface materials could consist of hemicelluloses, lignin, pectin, wax, and other impurities [31]. In Figure 5, it can be seen that these materials were partly removed due to 2 wt.% NaOH treatment. This observation proved that NaOH solutions could strip the surface substances of bamboo fibers. Furthermore, the surface materials were completely dissolved after stronger NaOH treatment, as shown in Figure 6. Meanwhile, we could see that a single bamboo fiber was actually made up of many elementary bamboo fibers, and the surface of the fiber became rough due to the removal of the surface materials. Accordingly, the rough surface could be expected to improve the interfacial bonding between bamboo fibers and epoxy matrix by mechanical interlocking. With regards to 10 wt.% NaOH treatment, bamboo fibers were severely corroded, resulting in considerable damage to the surface. Additionally, severe fiber fibrillation was introduced by the effect of high concentration of NaOH solution, which compromised the integrity of the fiber bundles. According to Ref [32], the decrease in strength for higher alkali concentrations can also be attributed to the partial removal of cellulose. Hence, excessive alkali treatment caused damage to the mechanical properties of a single bamboo fiber, and was deemed unsuitable for treating bamboo fibers. To conclude, 6 wt.% NAOH treated bamboo fibers were more suitable for fabrication of bamboo fiber/epoxy composites, which was also supported by single fiber tensile test.

### 3.2. FT-IR Analysis

**Figure 8:**
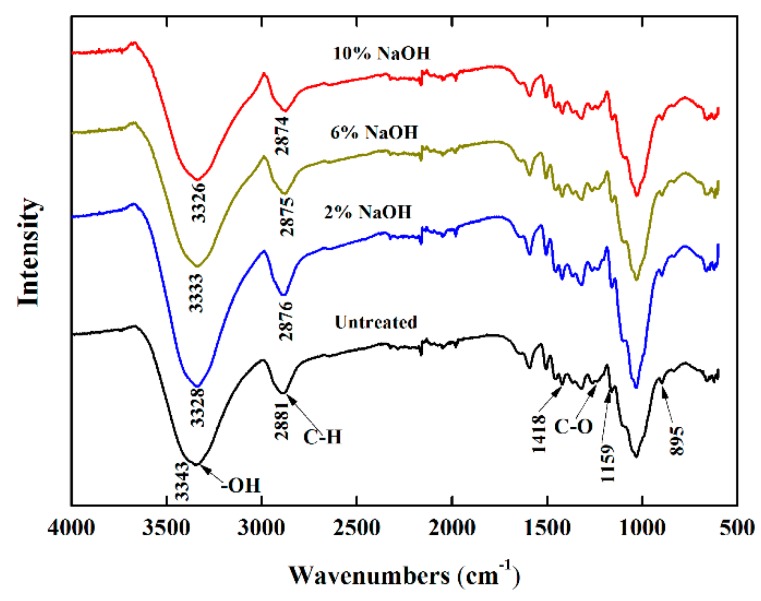
FT-IR spectra of untreated and 2–10 wt.% NaOH treated bamboo fibers.

The chemical structures of untreated and 2–10 wt.% NaOH treated bamboo fibers were studied by FTIR. As shown in Figure 8, the FTIR spectra were offset vertically by Software Oringin for clarity.

The FTIR spectra of all samples showed typical spectra of cellulose and were in line with previous studies [16,33]. The peaks at about 3343, 2881, 1159, and 895 cm^−1^ are attributed to the –OH, C–H, C–O and C–OH, respectively [34,35,36]. On the whole, the FTIR spectra for all samples were nearly identical, indicating that no new functional groups were added in the cellulose molecules. According to Ref. [37], as a kind of herbaceous plants, the basic tissue of bamboo fibers consist of epidermal, vascular, and hypodermis; the vascular bundles indicate so-called *bamboo fiber bundles*. When bamboo fibers were treated using a dilute alkaline solution, only the surface materials could be partly removed. Hence, simple alkali-treatment could not change the internal chemical constituents of fiber bundles, in spite of changing the surface morphology to some extent. However, compared to untreated fibers, the locations and intensity of characteristic peaks for alkali-treated fibers show a difference. The –OH stretching vibration absorption peak of 2–10 wt.% NaOH treated bamboo fibers had a right shift by 15, 10, and 17 cm^−1^, while the C–H stretching vibration absorption peak shifted from 2881 cm^−1^ to 2876, 2875, and 2874 cm^−1^, respectively. The peak observed at 1418 cm^−1^ can be ascribed to the CH_2_ deformation in cellulose. The peak at 1233 cm^−1^ can be readily assigned to the C–O stretching vibration of acetyl groups in lignin. The difference among FTIR spectra could be associated with the dissolving of the surface substances of bamboo fibers after alkali treatment. The scenario was supported by SEM analysis (see Figure 5, Figure 6 and Figure 7), which showed the non-structural materials, such as hemicellulose, lignin, pectin, and other impurities were partly removed after alkali treatment [17].

### 3.3. Mechanical Properties of Single Bamboo Fiber

The mechanical properties of bamboo fibers are affected by many factors, such as growth environment, growth years, and fiber extraction method [26]. Therefore, it is necessary to measure the mechanical properties of bamboo fibers used in this study. Considering the severely unstable mechanical properties of bamboo fibers, a single fiber tensile test was carried out for 20 specimens to achieve a valid average.

**Figure 9:**
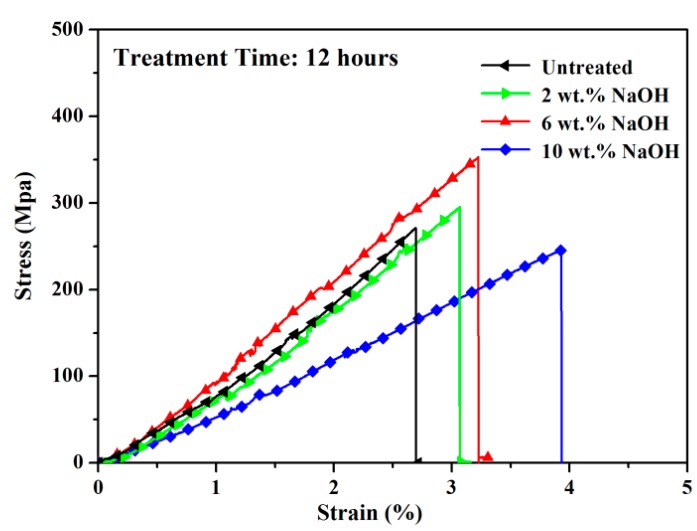
Typical Stress-Strain Curves for untreated and 2–10 wt.% NaOH treated bamboo fibers.

The results were summarized in Figure 9 and Table 1. The untreated bamboo fibers had an average tensile strength of 262 MPa and a Young’s modulus of 9.8 GPa. Compared to untreated fibers, 2 wt.% NaOH treatment had a minor effect on the tensile properties of bamboo fibers. As shown in Figure 5, 2 wt.% NaOH treatment could only remove small part of the surface substances, indicating that a large amount of gummy material still existed as a constraint among cellulose chains. During the tensile test, the internal constraint would prevent the fibrils from rearranging themselves in a compact manner along the direction of force. Hence, 2 wt.% NaOH treatment was not sufficient to improve the tensile strength of bamboo fibers.

However, after 6 wt.% NaOH treatment, the tensile strength and the Young’s modulus of bamboo fibers increased by 38% and 14%, respectively. According to Refs. [38,39], moderate alkali-treatment could effectively remove the hemicellulose and lignin in bamboo fibers, so that cellulose crystallinity increased, which usually improved both fiber tensile strength and modulus. For higher concentrations of NaOH treatment, the bamboo fibers remained a certain tensile strength, but there was a significant decrease in Young’s modulus, which was also found in other relevant studies [17,40].

### 3.4. Interfacial Shear Strength Measurement

**Figure 10:**
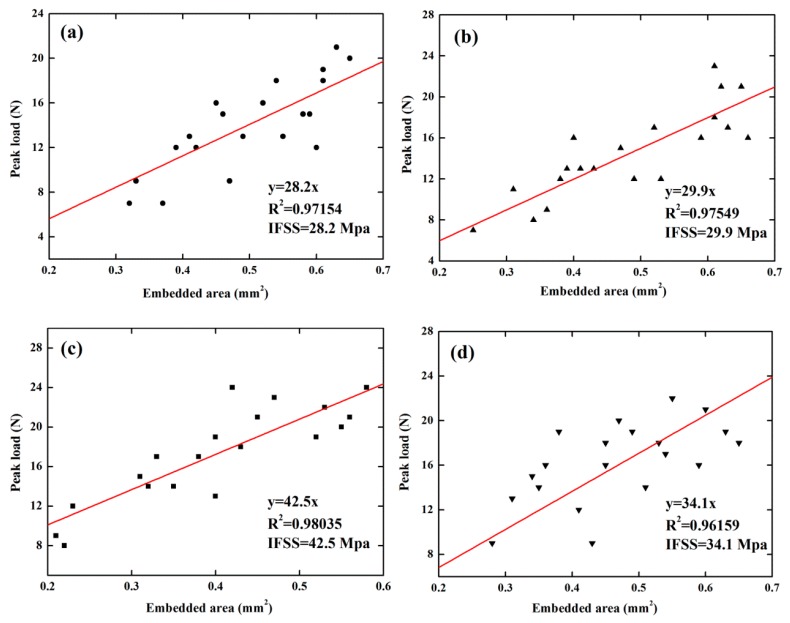
IFFS results: (**a**) untreated bamboo fibers, (**b**) 2 wt.% NaOH treated bamboo fibers, (**c**) 6 wt.% NaOH treated bamboo fibers, and (**d**) 10 wt.% NaOH treated bamboo fibers.

Compatibility between natural fibers and resin matrix is one of the decisive factors affecting the mechanical and thermal properties of composites. Hence, in this research, we studied the interfacial adhesion between the bamboo fibers and the epoxy matrix by a commonly used single fiber pull-out test. As shown in Figure 10, discrete data points about peak load and embedded bamboo fiber surface area were linearly fitted by Software Oringin. It was evident that 6 wt.% NaOH treated bamboo fibers exhibited better interfacial shear strength, which was in line with SEM observations (see Figure 6). However, compared to untreated bamboo fibers, other alkali-treated fibers did not show a significant increase in IFSS values.

**Figure 11:**
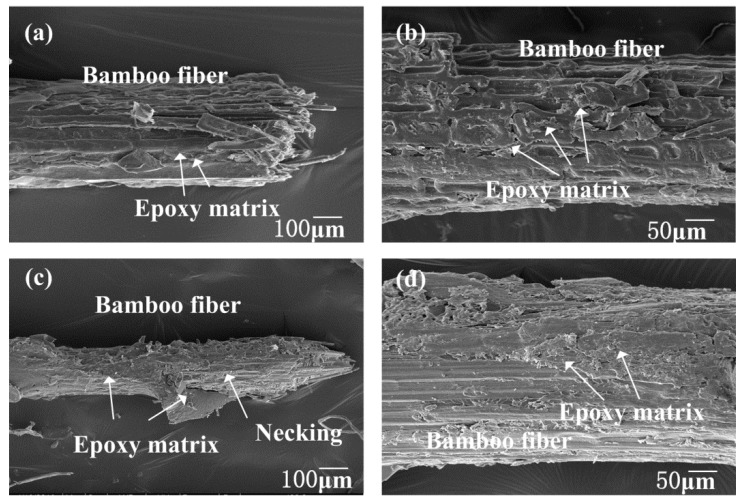
SEM images of bamboo fiber-reinforced epoxy after pull-out tests: (**a**) untreated bamboo fiber, (**b**) 2 wt.% NaOH treated bamboo fiber, (**c**) 6 wt.% NaOH treated bamboo fiber, (**d**) 10 wt.% NaOH treated bamboo fiber.

In order to study the effect of different fiber pretreatments on the interfacial adhesion between bamboo fibers and epoxy, SEM was used to observe the surface of fibers after a pull-out test. As shown in Figure 11a, little epoxy resin adhered to the surface of the untreated bamboo fiber after the fiber pull-out test, indicating poor interfacial adhesion between fiber and epoxy. This was largely because untreated fibers had many hydrophilic substances on the surface, and a relatively smooth and irregular surface morphology (confirmed by Figure 3). In Figure 11b,d, 2 wt.% and 10 wt.% NaOH treated fibers became more compatible with the resin, and more epoxy could be seen on the fiber surface. Interestingly, from the Figure 11b, 6 wt.% NaOH treated fibers were hard to pull out, and showed obvious necking during the test. This was attributed to the clean and rough surface morphologies after moderate alkali treatment. This phenomenon proved that 6 wt.% NaOH solutions could effectively improve the interfacial boding between bamboo fibers and epoxy matrix.

### 3.5. Thermal Properties

Unlike synthetic fibers with excellent thermal properties such as glass fiber and carbon fiber, the poor thermal stability of plant fibers is considered to be one of the limiting factors that restrict its further application.

**Figure 12:**
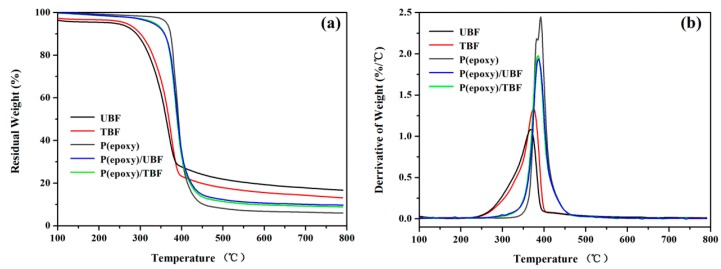
TGA (**a**) and DTG (**b**) curves of untreated and 6 wt.% NaOH treated bamboo fibers, neat epoxy and their composites.

The TGA and DTG curves for UBF, TBF, P(epoxy), P(epoxy)/UBF and P(epoxy)/TBF are depicted in Figure 12. Table 2 summarizes the detailed T_5%_, T_10%_, T_max_, and Y_c_ values, where UBF, TBF, P(epoxy), P(epoxy)/UBF, and P(epoxy)/TBF represented untreated bamboo fibers, 6 wt.% NaOH treated bamboo fibers, neat epoxy, composites reinforced with untreated bamboo fibers, and composites reinforced with 6 wt.% NaOH treated bamboo fibers, respectively. Between 40 and 105 °C, there was a tiny change on residual weight for neat epoxy and composites. However, 4% and 3% weight reduction occurred for UBF and TBF, respectively, which could be easily be ascribed to the vaporization of water in the fibers [25,36]. According to Ref. [41,42], the weight loss for bamboo fibers in the temperature ranges of 200–330 °C, 330–356 °C and 356–450 °C were mainly caused by the decomposition of hemicellulose, cellulose, and lignin, respectively. After 6 wt.% NaOH treatment, bamboo fibers had a Y_c_ value of 13.1%, while untreated fibers exhibited a bigger char yield of 16.7%. This was because NaOH solutions dissolved some of the lignin in fibers, which was deemed as the most difficult component to decompose [25]. Likewise, P(epoxy)/UBF had a Y_c_ value of 9.7%, while P(epoxy)/TBF presented a smaller value of 8.9%. In Figure 12b, the highest DTG peaks for UBF and TBF were located at 368 and 374 °C, respectively. However, the TBF (1.335 wt.%/°C) displayed 23.3% higher rate of DTG than UBF (1.082 wt.%/°C). In addition, there was only a minor change in T_5%_ and T_10%_ values between P(epoxy)/UBF and P(epoxy)/TBF. Although composites decomposed at lower temperatures compared to neat epoxy, bamboo fiber/epoxy composites still had a good thermal stability. Therefore, bamboo fiber composites fabricated in this study have potential for engineering applications.

### 3.6. Flexural Properties of Bamboo Fiber Reinforced Epoxy Composites

**Figure 13:**
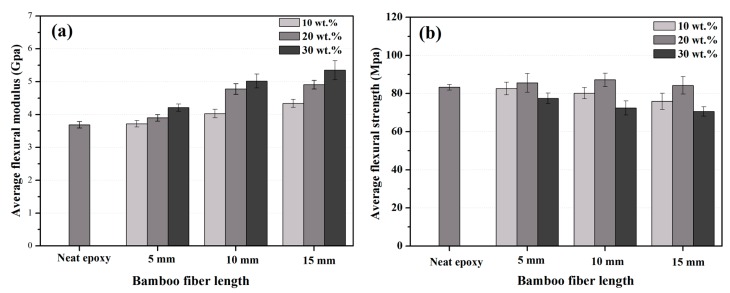
Average flexural strength (**a**) and modulus (**b**) of bamboo fiber composites compared to the neat epoxy.

**Figure 14:**
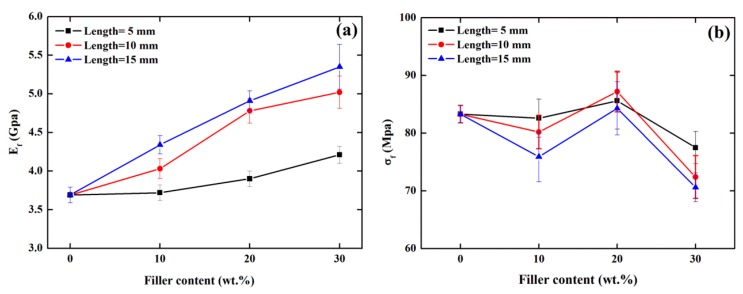
Filler length and content effect on the flexural modulus (**a**) and strength (**b**) of bamboo fiber composites.

The flexural test results, including flexural strength $(\sigma_{f}$) and modulus $(E_{f}$) of all samples were plotted in Figure 13 and Table 3, while Figure 14 presented the filler length and content effect on the flexural properties of composites.

When compared to neat epoxy, the flexural modulus for bamboo fiber composites were always higher, and monotonically increased with fiber length and content. This outcome was expected. According to Ref. [40], natural fibers have better mechanical properties, such as modulus, stiffness, and flexibility, than epoxy matrix. Meanwhile, it is easy to understand that higher fiber content and fiber length could provide higher bending stiffness during the flexural test. The average flexural modulus $E_{f}$ of neat epoxy was 3.69 Gpa, and the corresponding values for EP/BF_30_5, EP/BF_30_10, and EP/BF_30_15 were increased by 18%, 33%, and 45%, respectively. However, for all samples, composites showed negligible differences in flexural strength. This result indicated that the bamboo fibers did not act effectively as reinforcements to the epoxy resin. The flexural strength of bamboo fiber/epoxy composites was still controlled by the matrix properties. This was mainly attributed to the fact that bamboo fibers were randomly oriented in the epoxy matrix [6]. The decrease of $\sigma_{f}$ value for BF/EP_10_5-15 could be explained by the fact that the low content fibers cause non-uniform distribution, thus acting as potential defects, resulting in the decline of flexural strength. For BF/EP_30_5-15, as the bamboo fiber content increased, epoxy could not effectively penetrate the bamboo fiber bundles, and the possibility of fiber agglomeration was greatly increased, which was detrimental for the macroscopic, mechanical properties of the composites. However, the flexural strength for randomly oriented bamboo fiber reinforced epoxy composites (at a content of 20 wt.%) showed a slight increase, which was rarely seen in previous experimental studies [43,44]. This could be due to the special technology used in this study to prepare composite materials. The preparation process of composites greatly improved the interfacial bonding between bamboo fibers and epoxy matrix, and reduced the internal defects of materials, thus improving the flexural strength of resulting composites. It is worth mentioning that the thermal and mechanical properties of polymer blend were largely determined by the compatibility between two different components [45,46].

### 3.7. Fracture Properties of Bamboo Fiber Reinforced Epoxy

**Figure 15:**
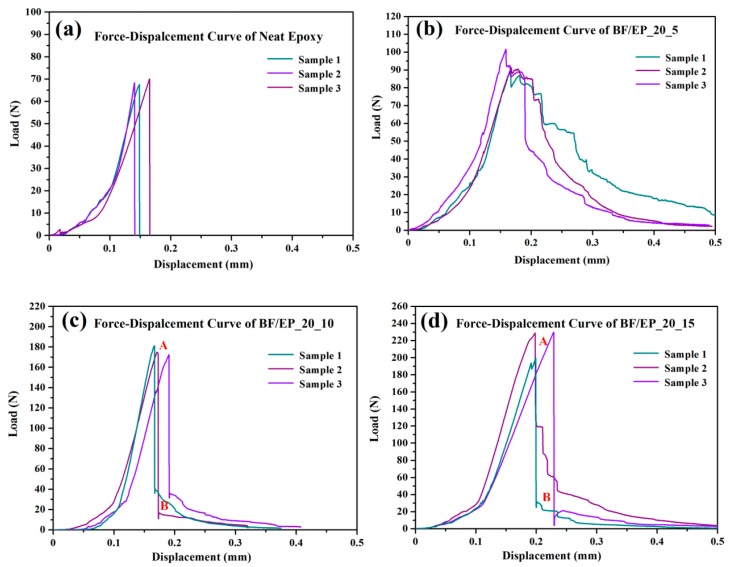
Force-displacement curves of bamboo fiber composites.

Representative load-displacement curves obtained from quasi-static fracture tests on neat epoxy and BF/EP_20_5-15 are depicted in Figure 15. BF/EP_20_5-15 were selected due to the relatively high flexural strength.

**Figure 16:**
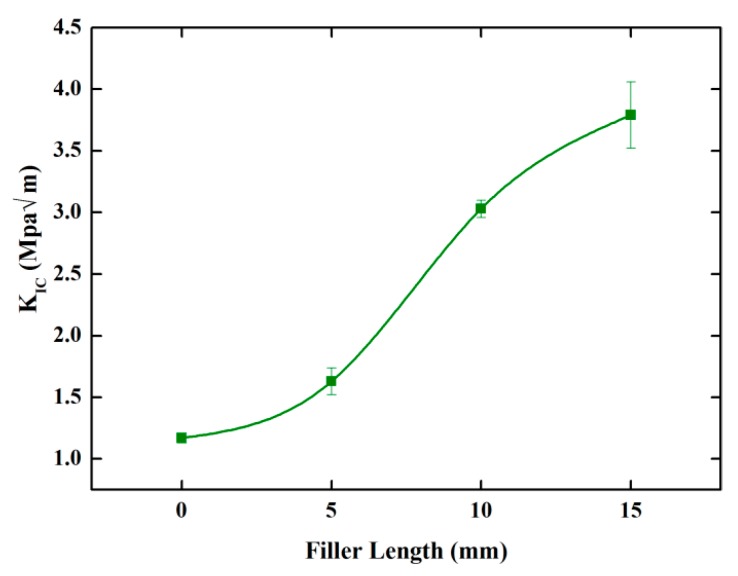
Filler length effect on the fracture toughness of bamboo fiber composites.

Before the fracture initiation, all the samples showed a near linear elastic behavior. From Figure 16, it was evident that the fracture properties of composites were improved with the increase of fiber length. The initial fracture toughness values (K_IC_) were tabulated in Table 2. In Figure 15a, for the neat epoxy, the load had a near-linear increase with the increase of displacement. Then, a single and abrupt drop was observed after the load reached the maximum value, which was commonly observed in thermosetting resins [47]. During the fracture toughness tests, composites made the obvious sounds of fiber breakage when the crack propagated inside. From Figure 15b, the load-displacement curves for BF/EP_20_5 were very interesting, and obviously different from others. BF/EP_20_5 did not fail abruptly, and showed extensive stable crack growth. This was likely because shorter bamboo fibers could be dispersed more uniformly in the epoxy matrix, and corresponding composite materials are less defective; nonetheless, further study is needed. In contrast, in the cases of BF/EP_20_10 and BF/EP_20_15, when the initial crack began to expand in the composites, there was a huge drop in the load from point A to point B, shown in Figure 15c,d. Unlike neat epoxy, composites could still bear the load for a while. Manalo et al. [6] mentioned that the presence of bamboo fibers could provide significant resistance to crack growth, thus changing the overall failure mode from totally brittle to ductile, to some extent.

### 3.8. Fracture Surface Morphology of Fracture Toughness Test Samples

**Figure 17:**
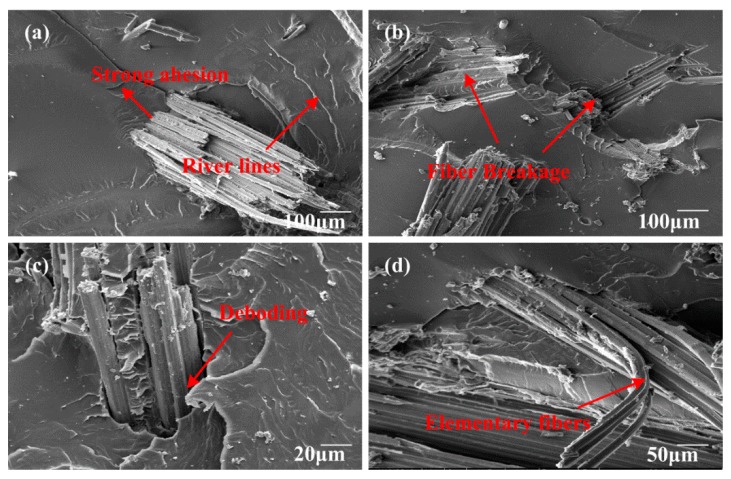
SEM images of composites with fiber length of 5 mm.

**Figure 18:**
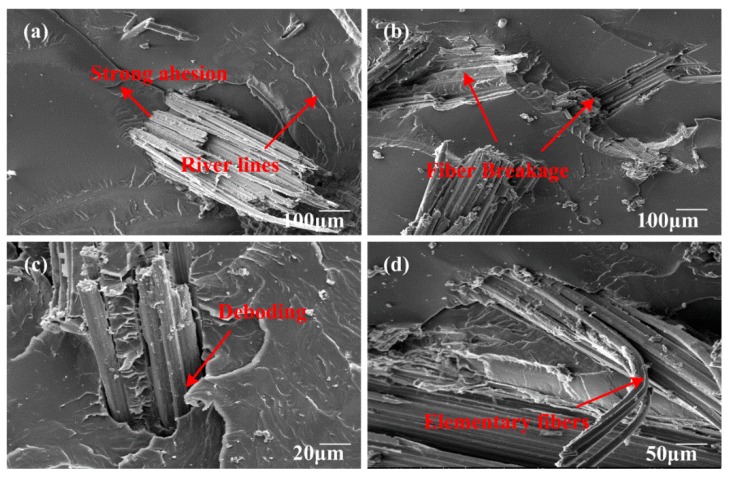
SEM images of composites with fiber length of 10 mm.

**Figure 19:**
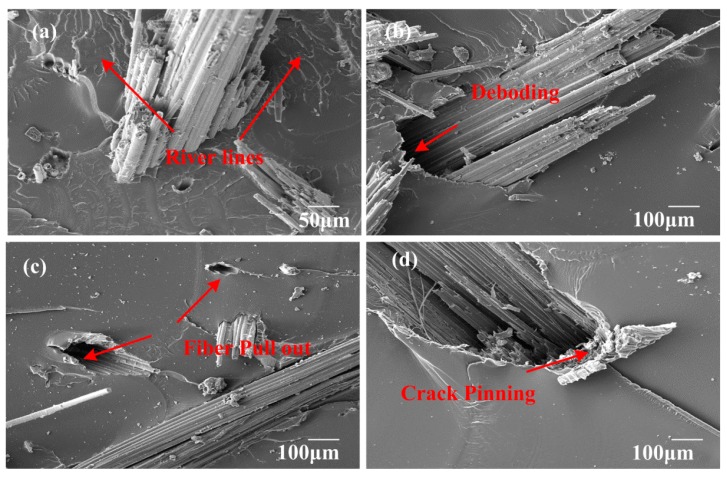
SEM images of composites with fiber length of 15 mm.

Figure 17, Figure 18 and Figure 19 show the SEM images of fractured surfaces. Obviously, different failure modes acted together and enhanced the fracture toughness of the composites. In Figure 17a, after moderate alkali treatment, bamboo fibers became more compatible with the epoxy, and were deeply embedded in the matrix. River lines were marked using a red arrowhead, which were often observed in fracture surface of brittle thermosetting resin. For EP/BF_20_5, fibers were hard to pull out; hence, fiber breakage and slight deboding between bamboo fibers and epoxy matrix contributed to the fracture toughness of the composites. Meanwhile, from Figure 17d, a fibrillation process took place; this could also consume energy to some extent, by mechanical friction, among elementary fibers. However, for EP/BF_20_10, fiber breakage and matrix cracks were dominant in the composites, as shown in Figure 18. According to Ref. [48], the interfacial adhesion between fibers and matrix was so strong that matrix cracking occurred around the stretched fiber. Unfortunately, despite great efforts to eliminate the entrapped air during the fabrication process, some bubbles were still found, as shown in Figure 18a. The existence of these bubbles could impair the macroscopic mechanical properties of bamboo fiber composites, which need further study. SEM images for EP/BF_20_15 were presented in Figure 19. It was evident that longer fibers were easier to pull out, and severe debonding between fibers and the matrix were also observed. These failure mechanisms can effectively create new superficial areas, thus enhancing the fracture toughness of composites. As shown in Figure 19b, crack pinning was another type of failure mode.

To conclude, several different reinforcing mechanisms acted together, and it was hard to quantify the predominant failure mode. Meanwhile, it is worth mentioning that Silva et al. [47] argued that the improved interfacial adhesion between randomly-distributed short-length fibers and the resin matrix impaired the main energy absorption mechanisms, thus having a negative effect on the fracture behavior of the composites. However, that discussion is not included in this paper.

## 4. Conclusions

In this study, 6 wt.% NaOH treated bamboo fibers were proven to be optimal for the fabrication of bamboo-fiber composites by a single fiber tensile test, single fiber pull-out test, Fourier transform infrared spectroscopy (FTIR), and scanning electron microscopy (SEM). Experimental study on flexural, fracture, and thermal behavior of bamboo fiber reinforced epoxy composites was carried out. The following are the main conclusions drawn from this study:

1. After 6 wt.% NaOH treatment, the tensile properties of bamboo fibers were improved, and bamboo fibers showed strong bonding with the epoxy matrix.
2. When compared to neat epoxy, the flexural modulus of composites are always higher, and monotonically increase with fiber length and content. However, for all samples, composites showed negligible difference on the flexural strength.
3. Compared to neat epoxy, there was a 39%, 159%, and 224% increase in fracture toughness values for BF/EP_20_5, BF/EP_20_10, and BF/EP_20_15, respectively.
4. Bamboo fibers treated with 6 wt.% NaOH solutions had better thermal stability than untreated fibers. Bamboo-fiber composites, therefore, have potential for engineering applications.
5. Fiber breakage, matrix cracking, debonding, and fiber pull out were major failure types, but the predominant failure mode was hard to quantify.

## Figures and Tables

**Table 1:** Mechanical properties of bamboo fibers before and after alkali-treatment.

NAOH Concentration (wt.%)	Tensile Strength (Mpa)	Young’s Modulus (Gpa)	Strain at Break (%)
untreated	262 ± 75	9.8± 1.6	2.7 ± 0.7
2	283 ± 71	9.2 ± 1.3	3.1 ± 0.8
6	363 ± 103	11.2 ± 2.4	3.3 ± 0.5
10	235 ± 67	6.1 ± 0.9	3.9 ± 1.1

**Table 2:** TGA summary for bamboo fibers, neat epoxy and bamboo/epoxy composites.

Samples	$T_{5\%}$ (°C) ^1^	$T_{10\%}$ (°C) ^1^	$T_{\max}$ (°C) ^2^	$Y_{C}$ (%) ^3^
UBF	236	289	368	16.7
TBF	264	299	374	13.1
P(epoxy)	363	372	392	6.0
P(epoxy)/UBF	330	356	386	9.7
P(epoxy)/TBF	334	358	387	8.9

^1^ Decomposition temperatures at 5 and 10% weight loss. ^2^ Decomposition temperature at the maximum decomposition rate obtained by derivative TG (DTG). ^3^ Residual yield at 800 °C.

**Table 3:** The flexural properties of alkali-treated bamboo fiber reinforced epoxy composites.

Samples	Flexural Modulus $E_{f}$ (Gpa)	Flexural Strength $\sigma_{f}$ (Mpa)	Fracture Toughness K_IC_ (Mpa$\sqrt{m}$)
**Neat epoxy**	3.69 ± 0.10	83.3 ± 1.5	1.17 ± 0.04
**BF/EP_10_5**	3.72 ± 0.10	82.6 ± 3.3	--
**BF/EP_10_10**	4.03 ± 0.13	80.2 ± 2.9	--
**BF/EP_10_15**	4.34 ± 0.12	75.9 ± 4.3	--
**BF/EP_20_5**	3.90 ± 0.10	85.6 ± 4.9	1.63 ± 0.11
**BF/EP_20_10**	4.78 ± 0.16	87.2 ± 3.5	3.03 ± 0.07
**BF/EP_20_15**	4.91 ± 0.13	84.3 ± 4.6	3.79 ± 0.27
**BF/EP_30_5**	4.21 ± 0.11	77.5 ± 2.8	--
**BF/EP_30_10**	5.02 ± 0.21	72.4 ± 3.7	--
**BF/EP_30_15**	5.35 ± 0.29	70.6 ± 2.5	--
